# A Gelatin Hydrogel-Containing Nano-Organic PEI–Ppy with a Photothermal Responsive Effect for Tissue Engineering Applications

**DOI:** 10.3390/molecules23061256

**Published:** 2018-05-24

**Authors:** Mantosh Kumar Satapathy, Batzaya Nyambat, Chih-Wei Chiang, Chih-Hwa Chen, Pei-Chun Wong, Po-Hsien Ho, Pei-Ru Jheng, Thierry Burnouf, Ching-Li Tseng, Er-Yuan Chuang

**Affiliations:** 1 Graduate Institute of Biomedical Materials and Tissue Engineering Taipei Medical University and International Ph.D. Program in Biomedical Engineering College of Biomedical Engineering, Taipei Medical University, No. 250, Wuxing Street, Xinyi District, Taipei 110, Taiwan; mantoshbiotech@gmail.com (M.K.S.); batzayanyambat@gmail.com (B.N.); kiwi8502018@gmail.com (C.-W.C.); afachen@doctor.com (C.-H.C.); s0925135546@yahoo.com.tw (P.-C.W.); thburnouf@ IPHtwo@hotmail.com (P.-H.H.); m120104012@tmu.edu.tw (P.-R.J.); thburnouf@gmail.com (T.B.); chingli@tmu.edu.tw (C.-L.T.); 2 School of Biomedical Engineering, College of Biomedical Engineering, Taipei Medical University, No. 250, Wuxing Street, Xinyi District, Taipei 110, Taiwan; 3 Bone and Joint Research Center, Department of Orthopedics, Taipei Medical University Hospital, School of Medicine, College of Medicine, Taipei Medical University, No. 252, Wuxing Street, Xinyi District, Taipei 110, Taiwan; 4 Graduate Institute of Biomedical Electronics and Bioinformatics, National Taiwan University, Room 410, Barry Lam Hall, No.1, Sec.4, Roosevelt Road, Taipei 10617, Taiwan

**Keywords:** polypyrrole, photothermal, gelatin, nanomaterial, NIR

## Abstract

The introduction and designing of functional thermoresponsive hydrogels have been recommended as recent potential therapeutic approaches for biomedical applications. The development of bioactive materials such as thermosensitive gelatin-incorporated nano-organic materials with a porous structure and photothermally triggerable and cell adhesion properties may potentially achieve this goal. This novel class of photothermal hydrogels can provide an advantage of hyperthermia together with a reversibly transformable hydrogel for tissue engineering. Polypyrrole (Ppy) is a bioorganic conducting polymeric substance and has long been used in biomedical applications owing to its brilliant stability, electrically conductive features, and excellent absorbance around the near-infrared (NIR) region. In this study, a cationic photothermal triggerable/guidable gelatin hydrogel containing a polyethylenimine (PEI)–Ppy nanocomplex with a porous microstructure was established, and its physicochemical characteristics were studied through dynamic light scattering, scanning electronic microscopy, transmission electron microscopy, an FTIR; and cellular interaction behaviors towards fibroblasts incubated with a test sample were examined via MTT assay and fluorescence microscopy. Photothermal performance was evaluated. Furthermore, the in vivo study was performed on male Wistar rat full thickness excisions model for checking the safety and efficacy of the designed gelatin–PEI–Ppy nanohydrogel system in wound healing and for other biomedical uses in future. This photothermally sensitive hydrogel system has an NIR-triggerable property that provides local hyperthermic temperature by PEI–Ppy nanoparticles for tissue engineering applications. Features of the designed hydrogel may fill other niches, such as being an antibacterial agent, generation of free radicals to further improve wound healing, and remodeling of the promising photothermal therapy for future tissue engineering applications.

## 1. Introduction

Effective regenerative treatment by tissue engineering has been progressively achieved by the development of novel functional materials and production of bioscaffolds in association with living cells and growth factors [1]. The biocompatibility of new biomaterials and associated factors are vital tissue engineering requirements during their development and application.

Polypyrrole (Ppy) is considered one of the useful conductive polymeric materials because of its numerous unique features, for example, stability of the chemical structure and higher electrical conductivity in aqueous mixtures [2]. Because of various biological requirements, the biocompatibility of Ppy has been studied previously. Numerous cellular and animal studies findings verified that Ppy derivatives are biocompatible with living systems and are free of any noteworthy injurious or inflammatory responses after long-term use [3]. Chemically, polypyrrole is a polymerized form of aromatic pyrrole monomer rings [4]. The pyrrole monomer is soluble in numerous distinctive kinds of aqueous phases and in chemical solvents, but Ppy is hydrophobic owing to an inflexible complexed backbone, and thus lacks appropriate functions for further biological applications [5]. To produce Ppy polymers from pyrrole monomers, oxidation and other chemical or electrochemical procedures have been used [6]. Currently, nanobiomaterials are being used in biomedical fields due to their similarities to biomolecules in terms of smaller dimensions; interestingly, nano-based biomaterials show a greater intracellular internalization as compared with micron-dimensional particles, making them excellent candidates for targeted drug delivery systems, such as those working toward targeting cancer cells and other specific sites [7], thus making them useful for many biomedical applications based on the functionality of nanobiomaterials and their interactions with the surrounding tissues. Recently synthesized Ppy nanoparticles can provide better structural properties and electrical conductivity for biomedical applications. Furthermore, polyethylenimine (PEI) is one of the most popular cationic polymers used in delivery systems [8], and could be formulated with Ppy for the production of stable Ppy nanoparticles. However, the use of PEI has been limited because of its possibly high hemotoxicity, cytotoxicity, and slow biodegradability at high-molecular-weight levels, confirmed by in vitro studies [9]. Low-molecular-weight PEIs have found to have better biocompatibility if there are chemical modifications [10,11] made. When combined together, PEI and Ppy, following an chemical process, may result in a PEI–Ppy polymeric nanocomplex with conductive properties [12] for extensive applications in biomedicine. PEI can also form a bridge between the Ppy and other polymeric substances such as gelatin due to its cationic nature. Gelatin has been considered as a popular polymeric biomaterial for multipurpose scientific applications [13] such as gel/hydrogel fabrication [14]. Features of gel-state gelatin are typically established by the structural changes and thermal properties in the environment [15]. Gelatin is soluble above 35 °C, but upon dropping the temperature to below 35 °C, natural thermal-reversible hydrogels are produced [16]. In this gelling process, chains of gelatin experience a transformational coil-to-helix conformation, through which gelatin manages to reverse the structure of the triple helix of ptotein [17]. It was reported previously that due to their physicochemical properties, polymeric hydrogels mimic the native extracellular matrix in skin and exhibit a high water-retaining capability, which is necessary for faster wound healing [18]. Hydrogel-constructed bioscaffolds also need to display remodeling properties over time to integrate with tissue during the healing process, and bridge with the natural extracellular matrix (ECM) of the potential patients. Solid or rigid biomaterial implants could not easily fit the shape of the contact surface of the defect [19]. Thus, the development of a hydrogel with a reversibly transformable property to fit defect surfaces would be a necessary design requirement for tissue engineering.

In this work, a photothermally responsive gelatin hydrogel containing a polyethylenimine (PEI)–Ppy nanocomplex (NC) was developed, and its physicochemical features were elucidated using confocal laser scanning microscopy (CLSM), scanning electron microscopy (SEM), transmission electron microscopy (TEM), and differential light scattering (DLS); its chemical properties by FTIR; and its photothermal behaviors via a thermal couple and camera. Cellular interaction behaviors with fibroblast cells were observed via MTT assay and fluorescence microscopy. We anticipated that this photothermal hydrogel system would possess a near-infrared (NIR)-triggerable property to provide local hyperthermia generated by a PEI–Ppy conductive nanoparticle composite in a gelatin hydrogel scaffold in a controlled manner, as well as being able to be used as a functionally active hydrogel. An in vivo study was designed using Wistar rat full thickness excision model for evaluation of our hydrogel system regarding its wound healing potential and signs of toxicity.

## 2. Results and Discussion

### 2.1. Preparation of the Test Gelatin Hydrogel Containing the PEI–Ppy NC

Imitating the inherent construction and biochemical features of the extracellular matrix (ECM) is a vital necessity in the design of biomaterial scaffolds to support cells for tissue regeneration, differentiation, proliferation, and migration [20]. Over the past decade, researchers have attempted to develop implantable biomaterials for applications in tissue engineering with improved communication between test biomaterials and living cells, with specific features such as adequate elasticity, mechanical stiffness, wettability, and surface topography [21], as well as additional growth factors for tissue regeneration.

**Figure 1:**
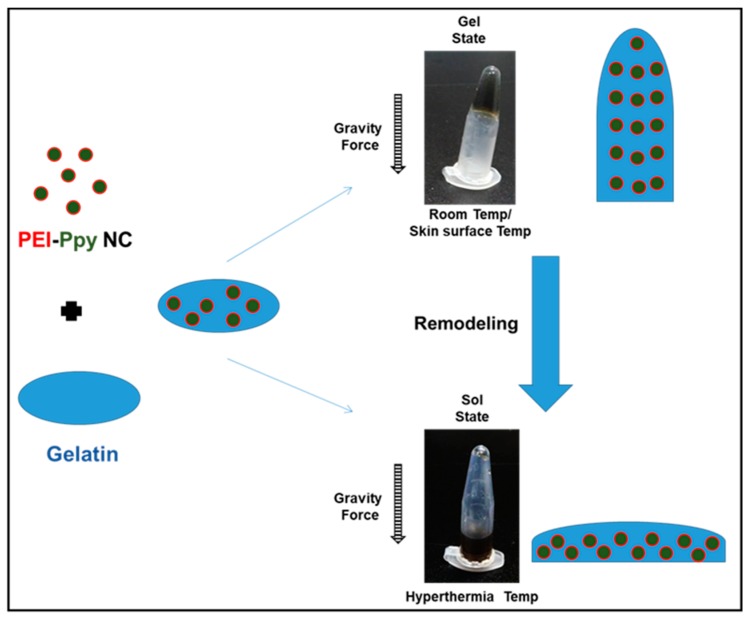
Schematic illustration of the fabrication process and reversible thermoplastic property of Polyethylenimine–Polypyrrole loaded gelatin hydrogel. Room temp. is around 22–25 °C, Hyperthermia temp. is around 39–45 °C. Temp: temperature.

As shown in Figure 1, the test PEI–Ppy NC was prepared using a nano-formation procedure similar to that described in a previous research work [22]. The test cationic PEI–Ppy NC exhibited a dark color on successful fabrication of Ppy nanoparticle. Next, the test cationic PEI–Ppy NC was mixed with a warm solution of thermoresponsive gelatin. The gelatin–PEI–Ppy‒NC composite hydrogel would be formed by ionic interaction between primary amines of the cationic PEI–Ppy‒NC with anionic carboxyl chains of the gelatin. After cooling down to room temperature, the gelatin hydrogel was formed against the force of gravity. The resulting photothermal hydrogel was found to have enhanced mechanical properties in the gel state at normal room temperature (Figure 1).

### 2.2. Physicochemical Properties of the Test Photothermal Hydrogel

When the photothermal hydrogel received a heat source, regional hyperthermia was developed, so that hydrogel inversely switched to a solution state. Gelatin in the melting state did not possess sufficient mechanical features to resist the force of gravity, such that the mixed solution was clearly observed to drop to the bottom of the Eppendorf tube (Figure 1). This may help in remodelling process of hydrogel after receiving remote NIR and is an advantage for covering defects of wound tissue.

As noted, physically triggered carrier systems have the ability to switch from a solution to a gel state. Thermally sensitive hydrogels, such as gelatin, employ thermal alterations by regulating the gelation process to improve biomedical applications [23]. The specific features of hydrogels enable them for biological uses, since they experience swelling ex vivo under certain physical circumstances and offer benefits for a suitable administration route. Gelatin with certain amounts of cationic PEI–Ppy NC may create stronger gels, since the covalent/ionic conjugation/bonding between polymeric chains would occur through the chemical reaction of the functional group with the corresponding reactivity, including those of isocynate hydroxyl group/amidogen or amine/carboxylic acid reaction [24]. Gels developed by using gelatin are thermally reversible at various physical temperature states. The gel–sol transitional behavior occurs at around 35 °C and above; that is, gelatin produces gel at temperatures of <35 °C and has a stable solution state at temperatures of >35 °C [25].

### 2.3. Size and Zeta Potential (ZP) of the Test Sample

**Figure 2:**
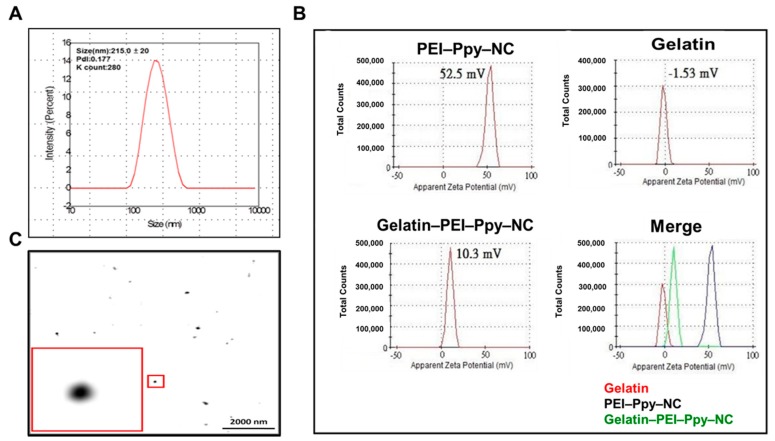
(**A**) Dynamic light scattering (DLS) size analysis of PEI–Ppy‒NC; (**B**) zeta potentials of the test samples PEI‒Ppy‒NC, gelatin, and gelatin‒PEI–Ppy‒NC; (**C**) TEM image of PEI‒Ppy‒NC with magnified view.

Size and zeta potential are prime important parameter analyses for the application of nanomaterials in any field. For design strategy, along with size, the positive surface charge of cationic polymers is a rationale for tissue regeneration. Previous studies focused on cationic modifications of polymer end-groups so that new biomaterial surface structures could be formed to facilitate tissue regeneration and for various types of wound management and healing applications [26]. Primarily, the size of the PEI–Ppy nanoparticle was found to be around 200 nm with 0.1 polydispersity index (PdI), as shown in Figure 2A, suitable for tissue engineering applications. To estimate the surface ZP of the test sample, the gelatin hydrogel with or without the PEI–Ppy NC was diluted 10-fold with PBS and then added into a ZP cuvette for further measurements. As shown in Figure 2B, charges of the PEI–Ppy NC before dialysis, gelatin, and gelatin–PEI–Ppy‒NC were determined to be +52.5, −1.5, and +10.3 mV, respectively. It was assumed that the positive charge was contributed by the cationic PEI polymeric amine, and the negative charge was donated by anionic carboxyl groups of gelatin for formation of the resulting composite hydrogel.

In the clinic, cationic hydrogels result in improved shrouding of living cellular retention in the infarcted position, leading to survival of attached cells gathered around the ischemic myocardium [27,28]. This feature of cationic hydrogels with positive surficial charge encourages cellular attachment and subsequent differentiation processes, and active end residues enable coupling to bioactive peptides. Characteristics of numerous natural and synthetic polymeric materials have been elucidated in hydrogel studies. Some candidates with cationic copolymers were previously reported as offering good hydrogel structural features. These polymeric materials have hydrophilic chemical groups which facilitate degradability through human proteases, thus affecting the biodegradability and biocompatibility, and have organic properties commonly required for biological strategies [29]. Cationic polymer-based hydrogels could possibly be engineered as bioscaffolds to achieve tissue repair. 

### 2.4. TEM Analysis of PEI–Ppy Nanoparticles

Nanoparticle size characterization by TEM is an important distinct analytical step before its application in diversified fields of biomedical sciences. The TEM image as shown in Figure 2C suggested that after the drying process, the nanoparticles (around 130 nm) have a uniform smooth circular structure which may be useful for potential biomedical applications by enhancing tissue growth and generating the stably uniform hyperthermia after NIR treatment.

### 2.5. Structural Morphology of the Test Photothermal Hydrogel

**Figure 3:**
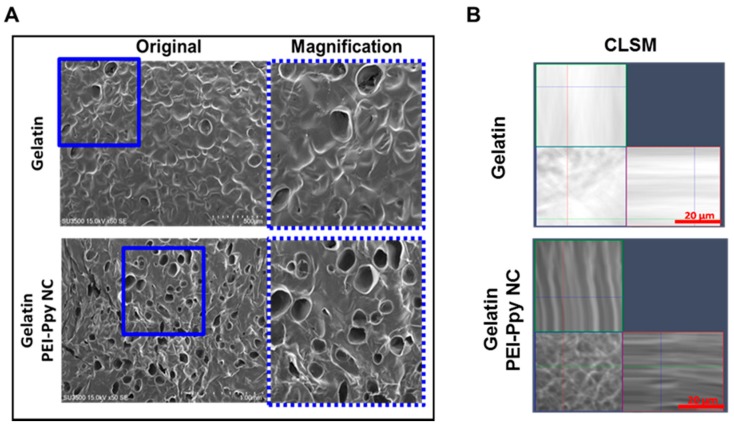
(**A**) Morphology and porous structure of the test sample detected by SEM: PEI–Ppy‒NC-loaded gelatin hydrogel (Gelatin, scale bar = 500 μm; PEI–Ppy‒NC-loaded gelatin, scale bar = 1 mm); (**B**) representative X–Z plane confocal laser scanning microscopy (CLSM) image of gelatin and gelatin containing PEI‒Ppy‒NC, showing the distribution of dark nanoparticles inside the gelatin hydrogel matrix.

Results of the morphological examination suggested that the porous structure of the test sample underwent lyophilization, as elucidated by SEM observations (Figure 3A). It was confirmed that a biomimetic scaffold exhibited a porous structural morphology that would be suitable for tissue engineering uses. The pore size was measured by an SEM method. The size distribution of the test hydrogel was around 0.1~0.2 mm. As noted in the literature, suitable pore sizes for living cells are in the range of 0.1~0.2 mm for cellular growth, proliferation, and regeneration [30].

Hydrogels can be applied in the field of tissue engineering either directly after their arrangement (without or with cellular entrapment) or after preparation as bioscaffolds. Hydrogel-based bioscaffolds are considered an extremely vital category of bioscaffolds, because of their ability to adapt their mechanical features to surrounding tissues and organs in the living system. Hydrogel bioscaffolds offer systemic and mechanical structures for living tissue construction, whether or not cells adhere to or are dispersed within the 3D porous structure of hydrogel networks. Furthermore, the uniform distribution of nanoparticles in the gelatin hydrogel matrix was observed by CLSM in the X–Z plane, as shown above (Figure 3B). This is an important characteristic feature of a stable hydrogel delivering nanoparticles at the defect site for healing and tissue regeneration.

**Figure 4:**
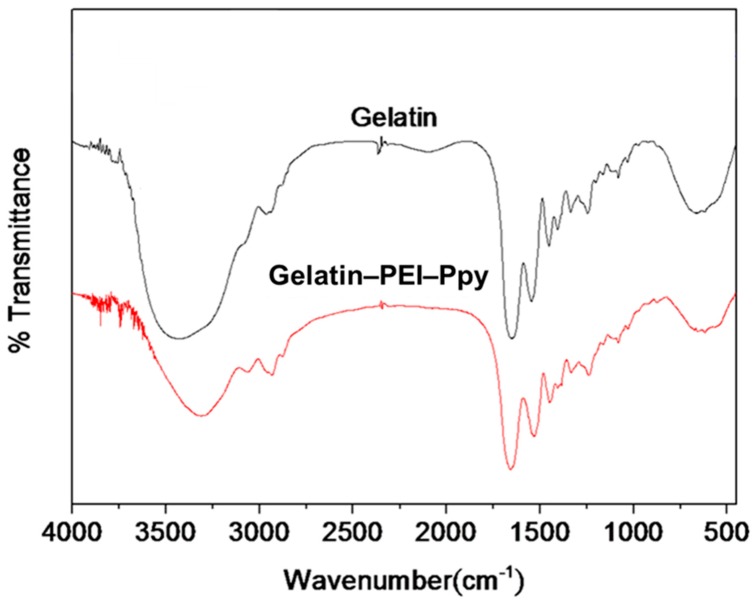
Results of the FTIR spectral analyses of the test samples of gelatin and the gel–PEI–Ppy NC hydrogel system.

Next, physicochemical characterization by FTIR revealed the detailed chemical interactions between the active ingredients of the composite hydrogel system. According to the results of the FTIR spectral analyses of the gelatin and gelatin‒PEI‒Ppy‒NC hydrogel samples (Figure 4), the band at 3400–3500 cm^−1^ corresponds to the NH groups in PEI–Ppy and gelatin. The signals at 2850–3000 cm^−1^ corresponds to the hydrocarbon stretching. The carbonyl stretch in the FTIR spectrum of gelatin was observed at 1650 cm^−1^. The other bands at 1200–1050 cm^−1^ correspond to –C–O and –C–N stretches. The chemical structure of gelatin contains carbonyl groups, and various aromatic and aliphatic amine groups are present in polyethyleneimine and polypyrrole. The presence of these polarized functional groups indicates a high probability of intermolecular chemical interaction, which is evidenced by the FTIR spectrum. Moreover, the presence of various hydrogen bond acceptors and donors also ascertains electrostatic interactions. The significant shift from 3400 cm^−1^ to 3200 cm^−1^, as observed from the FTIR spectrum of the physical mixture, could be due to the interaction of amine groups with the carbonyl group of gelatin. Hence, according to the results of the FTIR spectral analyses of the test samples of gelatin and the gelatin‒PEI‒Ppy‒NC hydrogel system, their corresponding chemical peaks were observed.

### 2.6. Photothermal Properties of the Test Hydrogel

**Figure 5:**
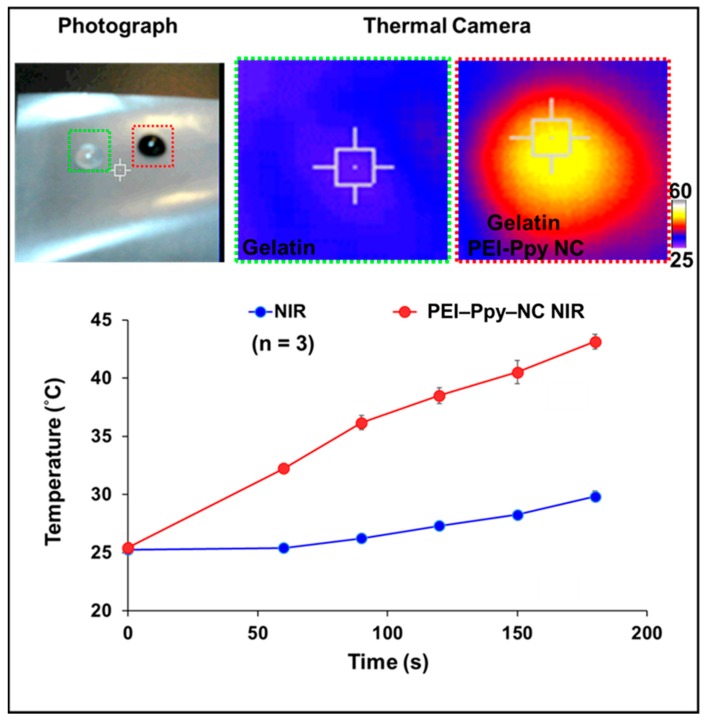
Results of the photothermal behavior of the gelatin‒PEI‒Ppy‒NC hydrogel treated by near-infrared (NIR) radiation and detected by a thermal camera (qualitative) or thermocouple for test PEI‒Ppy‒NC (quantitative, *n* = 3).

Photothermal behaviors after remote NIR irradiation were tested using a thermal camera. As a result, the thermal properties of the test hydrogel were clearly visualized, as shown in Figure 5. The temperature of the hydrogel without the PEI–Ppy‒NC was slightly elevated to 29 °C due to a lack of photothermal transduction efficiency. In contrast, after NIR treatment, temperature of the gelatin hydrogel with PEI–Ppy‒NC significantly increased to a hyperthermic temperature (43 °C) due to the presence of black Ppy conductive nanoparticles (Figure 5) [31].

Hyperthermic temperature is considered a treatment process to locally increase living tissue temperatures by modification of physical conditions [32]. Remote laser irradiation of photothermal materials has been used for the instantaneous hyperthermia used in medical fields [33]. Infrared thermography (IRT) imaging is considered to be a noncontact, noninvasive method for clinical diagnosis [34] which permits one to quantify and visualize IR radioactivity. IRT imaging has been used in clinics for a long period of time. Medical trials have impressive improvements in the area of tumors for analyzing malignant melanomas and breast cancer by IRT [35]. NIR has also been used as a thermal source in medicine, clinical imaging systems, and the treatment of various clinical problems [36]. Other applications may include the treatment of inflammatory arthritis, diagnosis of musculoskeletal injuries, locating tender points associated with fibromyalgia, evaluating microcirculation in vascular diseases, monitoring the repair of wounds after undergoing surgical processes, and treatment in syndromes of complex regional pain [37]. Physical state transformations such as sol–gel and various polymeric materials by external factors such as light and temperature (i.e., NIR) are one important rationale for the development of unique gel/hydrogel scaffold materials for drug delivery, wound healing, and various regenerative tissue engineering applications [38] at pathological target sites. In our study, the developed gelatin-based hydrogels, either containing or not containing active PEI–Ppy conductive nanoparticles, are solid at skin temperature (33.5 °C) [39]. At a hyperthermic temperature above the melting point (35 °C), due to remodeling of the viscous gel state, the developed photothermal hydrogel will melt to fit the defect surface area. Hence, this smartly designed formulation offers many advantages to assist wound healing and other regenerative processes in tissue engineering for clinical applications.

### 2.7. In Vitro MTT Cytotoxicity Assay

**Figure 6:**
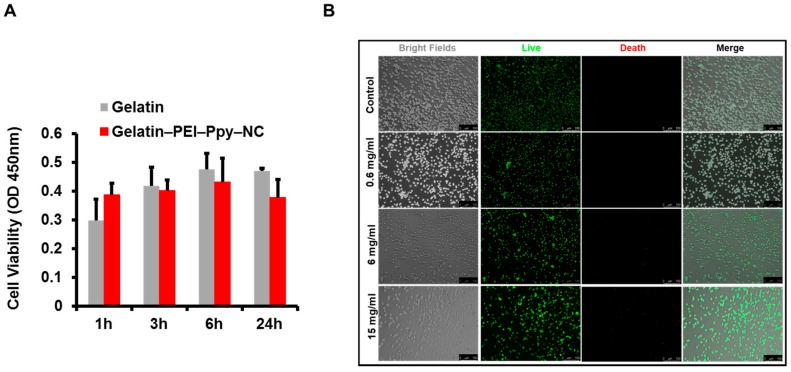
(**A**) Results of the MTT cytotoxicity assay of gelatin‒PEI–Ppy‒NC hydrogel (*n* = 3); (NS–non significant); (**B**) Results of cellular interaction behavior by live/dead staining test after incubating L929 cells at 37 °C, with the different amounts of PEI‒Ppy‒NC in gelatin hydrogel along with control group (gelatin only) detected by fluorescence microscopy. Scale bar‒100 µm. (OD: optical density)

According to the indirect MTT assay result, as shown in Figure 6A, in comparison to the control group of the gelatin-only hydrogel, the experimental groups containing various concentrations of PEI–Ppy‒NC were tested to check their effect on L929 fibroblast cell lines. Interestingly, PEI–Ppy‒NC nanoparticles in gelatin hydrogel showed similar biocompatibility to gelatin hydrogel groups. Furthermore, we could not find any significant cell viability difference between all the test groups up to 24 h of the experiment. To further support the MTT assay, we performed a live/dead assay to visualize the live and dead cells after cell and hydrogel interaction for 24 h.

### 2.8. Cellular Interactions with the Test Photothermal Hydrogel

When cells interact with hydrogel, favored by a suspension-like environment in the designed scaffold due to the presence of suitable polypeptide moieties on the superficial area of the hydrogel bioscaffold structure, the majority of the attached cells begin to proliferate and grow further [40]. Cell survival is further assisted by the arginine–glycine–aspartic acid (RGD)-like adhesive polypeptide structures in our test gelatin hydrogel. Finally, introduction of the RGD-like group in the test formulation revealed cell proliferation, growth, migration, and reorganization in the living tissue, supporting the tissue regeneration process [41]. Recent studies revealed that various cell types, for instance, smooth muscle cells, chondrocytes, endothelial cells, osteoblasts, and fibroblasts, can constructively attach to the functional polypeptide-adapted hydrogel bioscaffolds [42].

Cellular interactions with the test samples were examined using fluorescence microscopy, as shown in Figure 6B. The cationic photothermal gelatin hydrogel still exhibited a relatively higher amount of live cells similar to the gelatin-only hydrogel control group. According to the similar finding of a previous publication, cell biocompatibility with the test hydrogel would be associated with interactions between the cationic hydrogel and the anionic cellular surface [43]. The result confirmed that the designed hydrogel is a biocompatible material and may be used safely in tissue engineering and regenerative medicine. Each implant material has related adversities; thus, we need to strategize and develop an engineered biomimetic matrix or scaffold system that can function as a microarchitectural structure with adequate superficial space for cellular proliferation, cellular differentiation, cellular adhesion, and cellular migration of suitable living tissues [44]. Suitable hydrogel properties include biodegradation of engineered scaffolds, delivery of bioactive molecules, and cellular migration at the implanted site [45].

**Figure 7:**
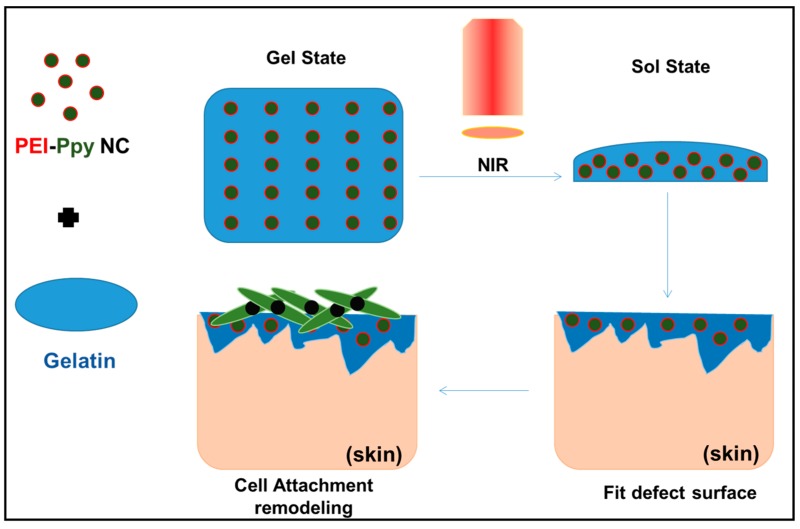
Schematic illustration showing application of the developed photothermal hydrogel (gelatin‒PEI‒Ppy‒NC) with thermoplastic properties after receiving NIR (melting) in skin tissue engineering applications.

In this study, the developed biomaterial hydrogel was found to have a photothermally reversible remodeling property during its exposure to NIR for the completion of remodeling for fitting the defected surface, and exhibited biocompatibility with cells (e.g., fibroblasts), which may enable it to be applied in the future in clinics to enhance the growth or repair of living tissues after suitable fitting at the defect site, as illustrated in Figure 7.

Tuning of the cell adhesion properties of hydrogel biomaterials for the growth of fibroblasts or osteoblasts has been insufficiently studied [46]. Additionally, cell attachment to positively charged materials was found to be more effective than that of polypeptide bioligands and neutrally charged and net negatively charged materials and molecules [47]. This management strategy on the basis of molecular charge advances the progress of new functional materials for improving or delaying the attachment of various types of cells such as fibroblasts or osteoblasts for the applied field of tissue engineering.

### 2.9. In Vivo Study Report

During the development of tissue engineering biomaterials for clinical usage, wound and wound healing studies are primary in vivo animal experiments required to be performed [48]. It implies different physiological processes are related to wound healing such as the proliferation of fibroblasts, anti-inflammatory activity, expression of fibroblast proteins, effect toward keratinocytes, antimicrobial activity, formation of collagen lattices, and antioxidant effects [49].

#### 2.9.1. Wound Size Contraction

**Figure 8:**
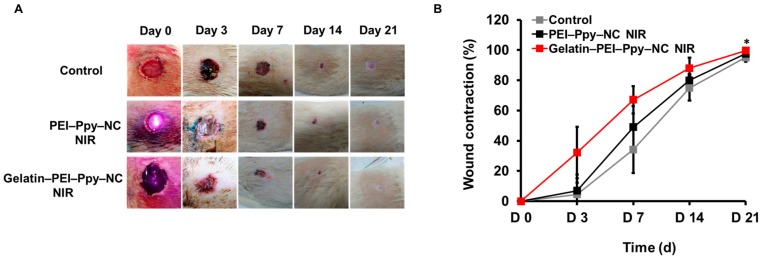
(**A**) Periodical wound healing evaluation (full thickness wound in the Wistar rat model): macroscopic images of the wound site and wound area of the control and two experimental groups at different time points (day (d) 0, 3, 7, 14, and 21) (*n* = 3); (**B**) Wound contraction (%) at various stages of wound healing and complete wound closure from day 0 to day 21(*n* = 3) (*p* < 0.05).

Prior to histological analysis of the wound site skin, the wound area and contraction were visualized, monitored, and captured as digital images, followed by periodical measurements at days 0, 3, 7, 14, and 21, as shown in Figure 8A. Then, the percentage of wound size contraction was calculated and graphically represented (Figure 8B) (*n* = 3) to evaluate the nature of wound healing in experimental groups such as the PEI‒Ppy‒NC NIR and gelatin‒PEI‒Ppy‒NC NIR groups, in comparison to the without-treatment control group. From macroscopic image observation, although at day 21, all groups of rats had shown almost complete wound closure, the gelatin‒PEI‒Ppy‒NC group showed the best results at days 3, 7, and 14. Moreover, there was significant difference between control and gelatin‒PEI‒Ppy‒NC NIR group at day 21 (*p* < 0.05) in terms of wound contraction percentage. Wound healing process in gelatin‒PEI‒Ppy‒NC NIR treated group had best healing without abnormal findings such as severe inflammation, necrosis, swelling, and infections due to biocompatibility of the test hydrogel.

#### 2.9.2. Wound Site Histology

**Figure 9:**
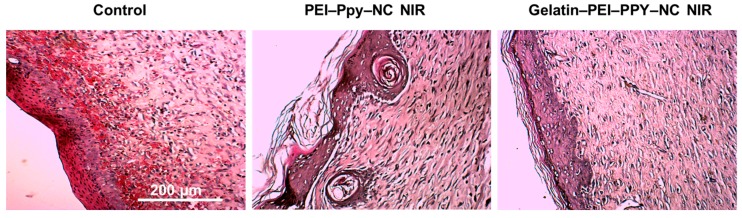
Histological findings from the untreated control and experimental groups’ wound site skin tissue at three weeks of wound healing.

Further confirmation of wound healing capacity and local toxicity of the materials implanted at the wound site were checked by detailed histological finding analyses, as shown in Figure 9. In general, re-epithelialization, granulation tissue formation, and fibrous tissue formation are the important characteristic phenomena, with appropriate cell migration and optimal cellular infiltration and organization within the wound site promoting the wound healing process [50]. In all three groups, during the experiment, they showed various degrees of re-epithelialization, regeneration, and repair with fibrous tissue formation. Re-epithelialization was found to be prominent at the wound edges at different maturation levels. The control group showed newly formed thicker epidermis layer without keratin, while the dermis layer had free red blood cell deposition with disordered collagen fibers and few inflammatory cells, which indicates altered wound healing [51]. In case of PEI‒Ppy‒NC NIR group, higher keratinization with irregular re-epithelialization was observed. But in the case of the gelatin‒PEI‒Ppy‒NC NIR treatment group, relatively normal re-epithelialization, fibroblasts, and organized collagen fiber formation were observed showing the biocompatibility and nontoxicity of the implanted scaffold material for wound healing and skin tissue engineering applications. Furthermore, our designed scaffold has the advantage to compensate for the toxicity of the PEI–Ppy NC at the wound site through the gelatin hydrogel scaffold system for full thickness skin wound healing.

#### 2.9.3. Body Weight Change and Organ Toxicity

**Figure 10:**
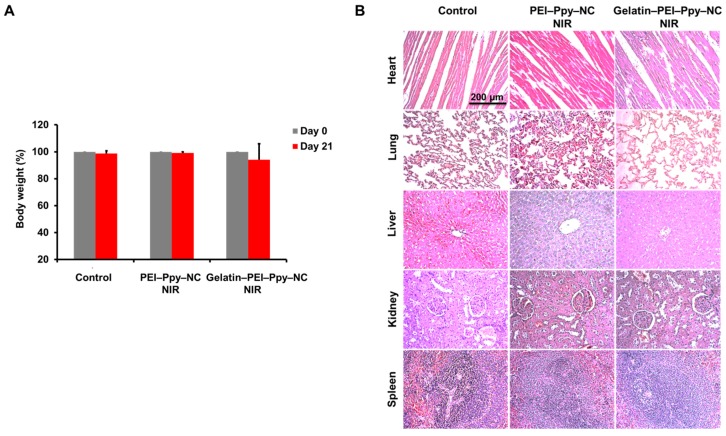
(**A**) Comparative body weight change (%) between day 0 and day 21 of the experiment in the control and experimental groups; (**B**) Histological findings of primary organs from the control and experimental Wistar rat groups’ heart, lung, liver, kidney, and spleen tissue.

There was no obvious significant body weight change observed among all the groups of rats, graphically represented as percentage change in body weight (Figure 10A) (*n* = 3) at the initial and final time points of the experiment, with the primary evidence of no severe pathological abnormalities developed during wound healing. To further check the impact of gelatin‒PEI‒Ppy‒NC hydrogel, systemic toxicity in different groups of Wistar rats was analyzed by gross as well as histological analyses which did not reveal any signs of inflammation and toxicity in control or experimental groups (Figure 10B).

## 3. Materials and Methods

### 3.1. Preparation of the Test Gelatin Hydrogel Containing PEI–Ppy‒NC

All test chemicals and kits were bought from Sigma-Aldrich (St. Louis, MO, USA) and were of analytical grade. Chemicals used in the cell investigations were purchased from Life Technologies (Carlsbad, CA, USA). The PEI‒Ppy‒NC was fabricated with a nano-formation technique similar to that described before [22]. Briefly, PEI (0.6 kDa, 0.2 g) was dispersed in deionized (DI) water (0.02 L) with magnetic stirring and then was blended with a formulated amount of the pyrrole monomer (12.5 μL). Next, this solution mixture was continuously stirred (at pH 0.8). Ferric chloride hexahydrate (0.0125 g/mL, 1 mL) was then added into the above aqueous phase experimental solution. After around 1 h of polymerization and solution mixture became black color, the liquid PEI–Ppy‒NC solution mixture was purified by dialysis process (MW: 3 KDa membrane), and then an oven dry was performed to obtain PEI–Ppy‒NC. To prepare the gelatin hydrogel containing the PEI–Ppy‒NC, the gelatin (type B, from bovine skin) was dissolved at a final concentration of 200 mg/mL [52] in warm phosphate-buffered saline (PBS) until it had cooled to room temperature, with a final concentration of PEI–Ppy‒NC of 15 mg/mL. The pure gelatin hydrogel without the PEI–Ppy‒ NC was used as the control group for further use, as per experimental requirement. The tested hydrogels are purified serval time by dialysis before use.

### 3.2. Physicochemical Properties of the Test Photothermal Hydrogel

Physicochemical properties of the test sample: The viscous characteristic was observed by performing an antigravity test imaged by a digital camera and visualization; the size and zeta morphology were detected with the use of DLS, transmission electron microscope, and scanning electron microscope. For PEI–Ppy‒NC size analysis by DLS, the test sample was diluted 7–10 times with PBS (1×), put in a quartz cuvette, and placed inside the instrument at optimal conditions. To test the zeta potential at the fluid state, the test sample was diluted tenfold with PBS, and then the zeta potential (ZP) was determined by dynamic light scattering (Malvern Instruments, Malvern, UK). Furthermore, the nanoparticle dispersion was ultrasonicated to separate the particles, and the most stable dispersion was chosen for TEM characterization. A drop of the stable solution was placed on the TEM customized grid and dried in a vacuum oven. Then, the grid was inserted in the TEM machine for analysis. For SEM analyses, all the test samples were freeze-dried, and samples were mounted onto stubs with a gold-coated approach. Then, the samples were observed through a SEM instrument. Furthermore, the bright field of 3D confocal microscopy imaging of the X–Z plane was used to analyse the gelatin matrix with or without PEI–Ppy‒NC. Test samples placed onto a confocal dish were estimated using CLSM to confirm the structural changes of tested sample. FTIR characterization was done for checking the chemical properties and interactions between the materials. FTIR samples were prepared by a freeze-drying process for the gelatin-only and gel–PEI–Ppy‒NC hydrogels. The FTIR spectral peaks of the samples were observed using a spectrometer and specific peaks were analyzed by Origin Pro 75 software (OriginLab, Northampton, MA, USA). To study the photothermal features, a water phase of the test sample at pH 7.4 was exposed to an NIR laser (808 nm) at a power density of 2 W·cm^−2^. Infrared thermographic frames were detected using a thermal camera (HuaZhi Electronic Technology Co., Ltd., Zhengzhou, China). Qualitative data of the photothermal results were acquired with a thermocouple. 

### 3.3. Cellular Interactions with Test Photothermal Hydrogel in In Vitro Study

#### 3.3.1. MTT Cell Cytotoxicity Assay

Equal volumes (200 µL) of gelatin and gel–PEI–Ppy NC hydrogel materials (PEI–Ppy NC, 15 mg/mL, *n* = 3) were incubated in 0.5 mL high-glucose DMEM in 96-well plates (37 °C, 5% CO_2_) with humidified air for 24 h. 10 µL of the liquid extraction medium was taken for indirect MTT cytotoxicity assay. The L929 mouse fibroblast cells (5000 cells/well, 0.1 mL) were seeded into 96-well plates and were then cultured for around 24 to 48 h for appropriate attachment with growth. Then, extraction medium from hydrogel (each 10 µL) was added into each well and incubated for 24 h. At the end, cellular activity was checked using an MTT kit and a microplate photometer (Multiscan FC, MA, USA).

#### 3.3.2. Live/Dead Assay

Mouse skin fibroblasts, L929 (ATCC® CCL-1™) cell lines were kept in a regular cell growth medium (DMEM high glucose). The L929 cells were seeded onto the confocal dishes and kept with prepared samples as described in MTT assay at 37 °C in incubator for 24 h. Afterwards tested cells were rinsed three times with PBS and stained with a live/dead assay kit to elucidate cellular interactions with the developed hydrogel carrier system. In brief, the live/dead assay chemical kit (Molecular Probes) contains a dye for detecting dead cells (ethidium homodimer, Eth-D) and a dye for detecting live cells (acetoxymethyl ester of calcein, calcein-AM), formulated to working concentrations (4 and 2 µM, respectively, PBS). Prepared aqueous dye was added to the cells in confocal dishes for 0.5 h at room temperature. Fluorescent signals from live/dead cells were then visualized through a fluorescent microscopy (Olympus, IX81, Tokyo, Japan). The control group without treatment was also compared in this study.

### 3.4. In Vivo Study

Rat skin wound healing experiment has been used for several skin tissue engineering studies [53]. Thus, Wistar rat was primarily selected for skin wound model development.

#### 3.4.1. Ethics

The in vivo Wistar rat wound model experiment was performed after regulatory approval (LAC-2018-0130) by the Taipei Medical University institutional animal care and ethics committee. All surgical processes were carried out under inhalant anesthesia (isoflurane, 2–4%). The animals were sacrificed by euthanasia via an overdose of Zoletil and CO_2_ after finishing the experiment.

#### 3.4.2. Full Thickness Excision Wounds

Wistar rats were selected for this study. Full thickness wound models were developed. The body was shaved by a trimmer and aseptically cleaned prior to wound creation. After rats were anaesthetized and skin hairs on the back were removed, the skin was sterilized by using ethanol (70%), and next, a 20-mm diameter circle was drawn. A circular cut was made around the drawn surface area of skin; the skin was then carefully dissected to create a full thickness wound [54]. The wound of the excision region was recorded instantly. During the course of the experiment, tested Wistar rats were randomly divided into three equal studied groups (A, B, and C, *n* = 3). Group A was the control group (wound without any treatment), group B rats were treated with PEI–Ppy NPs with NIR immediately after wound creation, and group C rats were treated with gel–PEI–Ppy NC hydrogel with NIR at the wound site. All animals were left untreated by other means during the experimental periods. The study was conducted for 21 days, and the parameters assessed in each group (*n* = 3) were wound size, wound area and wound contraction (%). Animals were provided freely with food and water and were monitored periodically during the whole experiment for any or abnormal changes in them.

#### 3.4.3. Wound Area Observation and Percentage of Wound Contraction (Healing) Measurement

Change of the wound area was measured periodically during the study at days 0, 3, 7, 14, and 21. Wound contractions were monitored and calculated as wound contraction percentage by the following formula [55]:

W% = (W_d0_ − W_dn_)/W_d0_ × 100

where W_d0_: wound area at day 0 and W_dn_: wound area at day *n* (*n* = 0, 3, 7, 14, 21).

#### 3.4.4. Skin and Internal Organ Collection

After three weeks, the animals were sacrificed under anesthesia. Wound site skin, heart, lung, liver, kidney, and spleen were removed, fixed in 4% buffered formalin (Thermo Scientific, Schwerte, Germany), dehydrated using increasing concentrations of ethanol, and then embedded in paraffin. After sectioning, samples were stained with hematoxylin and eosin stain (as per standard staining procedure).

## 4. Statistical Analysis

All experimental data are expressed as the mean ± standard deviation. The data for each experimental group were examined by ANOVA. Statistical significance was estimated by using Graph Pad Prism 6 along with a statistical software package (IBM SPSS Statistics 20), with post-hoc multiple comparison and equal variance comparison by Scheffe test. *p* < 0.05 was considered to be statistically significant.

## 5. Conclusions

In this study, a photothermal gelatin hydrogel containing the PEI–Ppy‒NC was successfully prepared, and its intrinsic features were investigated by DLS, TEM, CLSM, SEM, and FTIR. Cellular interaction mechanisms of the test carrier system and the photothermal performance were studied. A morphological examination of the porous structure was carried out using SEM. Combined with the local hyperthermia acquired by NIR exposure, the photothermal triggerable/guidable hydrogel was structurally changed from a gel to solution state. Fibroblasts were shown to be biocompatible in the designed photothermal hydrogel with a cationic charge for further proliferation, migration, differentiation, and development. An in vivo study was conducted on a Wistar rat wound model to check the material compatibility with the living system, by considering the PEI–Ppy‒NC NIR treatment group and the gel–PEI–Ppy‒NC NIR-treated group. From this study, the gel–PEI–Ppy NIR-treated group was found to be a potent therapeutic strategy at the wound sites, showing progress made in the field of regenerative medicine regarding healing, tissue regeneration, and repair. Clinical application would be possible related with plastic surgery, orthopedics, and dental science by analyzing and controlling the growth patterns for healthy tissues versus pathological tissues with proper evaluation of the outcomes.
